# Antinuclear Antibodies Are Associated with an Increased Risk of Diffuse Large B-Cell Lymphoma

**DOI:** 10.3390/cancers15215231

**Published:** 2023-10-31

**Authors:** Eleanor Frost, Jonathan N. Hofmann, Wen-Yi Huang, Christine G. Parks, Ashley A. Frazer-Abel, Kevin D. Deane, Sonja I. Berndt

**Affiliations:** 1 Department of Epidemiology & Population Health, Stanford University School of Medicine, Stanford, CA 94305, USA; 2 Division of Cancer Epidemiology and Genetics, National Cancer Institute, Bethesda, MD 20892, USA; 3 Epidemiology Branch, National Institute of Environmental Health Sciences, National Institutes of Health and Department of Health and Human Services, Research Triangle Park, Durham, NC 27709, USA; 4 Department of Medicine, University of Colorado Anschutz Medical Campus, Denver, CO 80045, USA

**Keywords:** non-Hodgkin lymphoma, antinuclear antibodies, autoimmune biomarkers, immune dysregulation, diffuse large B-cell lymphoma, marginal zone lymphoma, chronic lymphocytic leukemia, nested case-control study

## Abstract

**Simple Summary:**

Some autoimmune diseases have been linked to an increased risk of non-Hodgkin lymphoma (NHL), but the evidence varies across different subtypes of NHL, and few studies have examined whether autoimmunity is more generally associated with disease risk. Given the rise in autoimmunity, as measured by antinuclear antibodies (ANA) over time in the U.S., it is important to evaluate its potential association with NHL risk. In this nested case-control study, we measured ANA and other autoimmune biomarkers in serum collected years prior to diagnosis for cases and controls. We demonstrate that the presence of ANA is associated with an increased risk of diffuse large B-cell lymphoma, a common subtype of non-Hodgkin lymphoma. We further show that specific autoimmune biomarkers are associated with an increased risk of NHL, especially diffuse large B-cell and marginal zone lymphoma. Our study establishes autoimmunity as a risk factor for diffuse large B-cell lymphoma.

**Abstract:**

Immune dysregulation is thought to increase the risk of non-Hodgkin lymphoma (NHL), but the evidence varies by subtype. We evaluated whether antinuclear antibodies (ANA), double-stranded DNA antibodies (anti-dsDNA), and extractable nuclear antigen antibodies (anti-ENA) were associated with the risk of common NHL subtypes in a nested case-control study. The autoantibodies were tested in serum collected years prior to NHL diagnosis in 832 cases and 809 controls from the Prostate, Lung, Colorectal, and Ovarian Cancer Screening Trial. Logistic regression was used to determine odds ratios (ORs) and 95% confidence intervals (95% CI) for the association with NHL risk. No association was observed between ANA positivity and NHL risk overall (OR: 1.18, 95% CI: 0.88–1.58); however, ANA positivity was associated with an increased risk of diffuse large B-cell lymphoma (DLBCL) (OR: 1.83, 95% CI: 1.15–2.91), with 19.7% of cases and 12.2% of controls testing positive. The presence of either anti-ENA or anti-dsDNA was associated with an increased risk of NHL (OR: 2.93, 95% CI: 1.18–7.28), particularly DLBCL (OR: 3.51, 95% CI: 1.02–12.0) and marginal zone lymphoma (OR: 8.86, 95% CI: 1.26–62.0). Our study demonstrates that autoantibodies are associated with an elevated risk of DLBCL, providing support for autoimmunity as a risk factor.

## 1. Introduction

Some autoimmune diseases, such as Sjögren’s syndrome, have been consistently associated with the development of lymphoid malignancies in epidemiologic studies [1]. Autoimmune disorders are more commonly reported among non-Hodgkin lymphoma (NHL) patients compared to controls [2]. A meta-analysis of 20 autoimmune disease cohort studies found that individuals with primary Sjögren’s syndrome, systemic lupus erythematosus, or rheumatoid arthritis had a significant increase in the risk of developing NHL compared to the general population, with standardized incidence rates ranging from 2.5 to 18.8 depending on the autoimmune disease [3]. Other autoimmune conditions, such as autoimmune hemolytic anemia, celiac disease, and psoriasis, have also been associated with an elevated risk of lymphoid malignancies in cohort and population-based studies [4,5]. For multiple myeloma (MM), a meta-analysis reported an increased risk among individuals with a history of any autoimmune disorder, particularly for those with detectable autoantibodies and with a history of autoimmune hemolytic anemia or pernicious anemia [6]. Among those with an autoimmune disease, disease activity and severity are risk factors for the subsequent development of lymphoma [7], suggesting that the disease process may be a contributing factor.

The increased risk associated with autoimmune disease appears to vary by both autoimmune and lymphoid malignancy disease type. Some NHL subtypes seem to be more commonly associated with autoimmune diseases than others [8]. Autoimmune diseases mediated by B-cell activation, such as Sjögren syndrome, have largely been associated with B-cell NHL subtypes, whereas T-cell activating autoimmune diseases, such as Crohn’s disease and ulcerative colitis, tend to be associated with an increased risk of T-cell lymphomas [8,9]. A pooled analysis found that B-cell activating autoimmune disorders were strongly associated with an increased risk of marginal zone lymphoma (MZL), diffuse large B-cell lymphoma (DLBCL), and lymphoplasmacytic lymphoma/Waldenström macroglobulinemia (LPL/WM), but less so with other subtypes [8]. Much of the evidence for autoimmune diseases and NHL risk, particularly for specific subtypes, comes from retrospective studies, where autoimmune disorders could have been identified as part of the diagnostic work-up and reverse causality is difficult to exclude.

Serum biomarkers can provide evidence of immune dysregulation years prior to symptoms or diagnosis of autoimmune disease. One biomarker of immune dysregulation and autoimmunity is the presence of autoantibodies, especially antinuclear antibodies (ANA). The prevalence of ANA in the U.S. population has increased over time and is estimated to be 13.8% based on an intensity of 3+ or higher at a 1:80 serum dilution level [10,11]. Although it is difficult to directly compare, the estimated prevalence of autoimmune disease in the U.S. and other Western countries is lower [12], suggesting that ANA may be more reflective of general autoimmunity. Indeed, ANA has been shown to have a low positive predictive value in predicting autoimmune disease in clinical settings [13,14]. ANA positivity is more common among older individuals and females [10]. ANA has also been related to environmental and occupational exposures, such as increased pesticide use [15] and trichloroethylene exposure [16], a suspected risk factor for NHL.

Retrospective case-control studies have reported a higher prevalence of ANA and other autoimmune antibodies among NHL cases than controls [17,18,19]. One study found that NHL patients had significantly higher levels of ANA using serum collected at diagnosis but before treatment for NHL [17]. A Swedish case-control study investigating immune markers in patients with lymphoid malignancy found significantly higher levels of autoantibodies in NHL patients [18]. Another study reported that many lymphoma patients testing positive for ANA did not appear to have clinical symptoms of autoimmune disease [20], suggesting that the presence of ANA, even in the absence of a clinical diagnosis or treatment of an autoimmune disease, may be a risk factor for NHL. As previous studies of ANA have been retrospective, it is difficult to determine if autoimmunity, as measured by ANA, is a risk factor for NHL risk or a consequence of the disease process.

We sought to investigate whether the presence of ANA was associated with an increased risk of developing NHL in a nested case-control study using serum collected years prior to NHL diagnosis. Our study included over 800 NHL cases, providing the opportunity to assess the risk of developing specific NHL subtypes, such as DLBCL, as well as B-cell and T-cell NHL.

## 2. Methods

Our nested case-control study comprised participants from the screening arm of the Prostate, Lung, Colorectal, and Ovarian (PLCO) Cancer Screening Trial. Nearly 155,000 individuals aged 55 to 74 years were recruited at 10 PLCO study centers in the United States from 1993–2001. Participants were actively followed for cancer diagnosis through 2009, with a median follow-up time of 11.3 years. Participants randomized to the screening arm of the trial completed a baseline questionnaire and had blood specimens drawn at baseline and annually for five years. Participants were actively followed for cancer incidence during the trial, and medical records were abstracted to confirm any reported cancer diagnosis. All participants provided informed consent, including consent for etiologic studies using stored biospecimens. The study was approved by the institutional review boards at the ten centers and NCI.

For this study, all participants were required to have completed baseline questionnaire, have available serum, and have had no diagnosis of cancer prior to blood draw. For cases, serum was required to have been collected prior to diagnosis. Cases (*n* = 832) included those diagnosed with a lymphoid malignancy, such as DLBCL, follicular lymphoma (FL), MZL, chronic lymphocytic leukemia (CLL), and multiple myeloma (MM), and were classified according to the InterLymph WHO hierarchical classification of lymphoid neoplasms [21]. Controls (*n* = 809) were required to be cancer-free at blood draw, alive at the time of case diagnosis, and have no diagnosis of hematopoietic or rare cancer during the trial. Controls were matched to cases in a 1:1 ratio on age at blood draw (+/− 1 year), race, gender, the calendar year of blood draw, and study year of blood draw (i.e., baseline for most). This study comprised 832 NHL cases and 809 controls. Cases included B-cell (*n* = 777), T-cell (*n* = 34), and unspecified (*n* = 21) NHL cases. B-cell NHL cases included diffuse large B-cell lymphoma (DLBCL, *n* = 147), follicular lymphoma (FL, *n* = 92), marginal zone lymphoma (MZL, *n* = 28), chronic lymphocytic leukemia (CLL, *n* = 223), and multiple myeloma (MM, *n* = 176) cases, among others.

### 2.1. Laboratory Analyses

This study utilized stored serum samples that were collected as part of the trial. As described previously [22], serum samples were aliquoted and stored at either −70 °C or −157 °C. All samples were obtained prior to NHL diagnosis, and laboratory analyses were performed in stored samples. Antinuclear antibodies (ANA) were measured using Hep-2 cell slides (Kallestad, Bio-Rad Laboratories, Hercules, CA, USA). Samples were incubated with a 1:80 dilution of sera, washed, and then incubated with the burro anti-human polyvalent immunoglobulin FITC conjugate (Kallestad). ANA fluorescence intensity was read using fluorescent microscopy (Leitz Fluorescence Scope, 50/1.0 magnification) and scored on a scale of 0–4 at increasing dilutions (e.g., 1:80, 1:160, 1:320). ANA positivity was established based on values of titer and intensity, with an intensity of 2+ at a titer of 1:320 considered positive for this study. Samples testing positive for ANA were subsequently tested for antibodies to anti-double stranded deoxyribonucleic acid (anti-dsDNA) and extractable nuclear antigens (anti-ENAs), including anti-Sjögren’s-syndrome type A (anti-SSA) and type B (anti-SSB) antibodies, anti-Smith (anti-Sm), and anti-ribonuclear protein (anti-nRNP). Anti-dsDNA was tested using BioRad Kallenstad indirect fluorescent antibody. Anti-ENAs were tested utilizing QUANTA Lite by Inova Diagnostics. Laboratory personnel were blinded to case-control status, and all assays were tested with positive and negative controls. Blind duplicates were included and yielded an average of 88.6% concordance within batches and 81.9% concordance across batches. As part of another study, we previously tested antibodies to cyclic citrullinated protein antigens (anti-CCP3) using the anti-CCP3 ELISA assay (Werfen, San Diego, CA, USA) and rheumatoid factor (RF) immunoglobulin M (RF-IgM) and A (RF-IgA) (Werfen, San Diego, CA, USA) using cutoffs for each assay established per laboratory protocols and recommended by the manufacture (i.e., ≥20 u/mL for anti-CCP3).

### 2.2. Statistical Analyses

We compared the baseline characteristics between NHL subtype cases and controls by implementing t-tests and chi-square tests with a Bonferroni correction to adjust for the number of tests (alpha level: 0.05/42 = 0.001). To test for the association between ANA and NHL risk, we ran logistic regression analyses utilizing ANA positivity as a binary variable to determine the odds ratios (ORs) and 95% confidence intervals (95% CI), adjusting for age, gender, and race. Variables, such as batch, smoking status, BMI, and NSAID use, did not significantly alter the ORs and were not included in the models. As other studies have reported substantial heterogeneity by NHL subtype, we examined the association with specific subtypes DLCBL, CLL, FL, MM, and MZL in logistic regression models in addition to B-cell NHL and T-cell NHL, adjusting for age, gender, and race. We conducted sensitivity analyses restricted to the smaller set of matched cases and controls, using both conditional and unconditional logistic regressions, which yielded similar results. Thus, results utilizing the full set of controls are presented. Stratified analyses were conducted based on age, gender, race, smoking status, BMI, the number of freeze thaws of the serum sample (1 or 2), years from blood draw to diagnosis (0–4 or 4+ years), self-reported autoimmune disease (ulcerative colitis, Crohn’s disease or rheumatoid arthritis vs. none), and rheumatoid arthritis-related antibody positivity (anti-CCP3, RF-IgM and RF-IgA). We evaluated potential effect modification of these same variables by including an interaction term in the logistic regression model and testing for significance. A global test of heterogeneity was utilized to determine whether the association with ANA differed across subtypes.

In addition to ANA, the risk of developing NHL, B-cell NHL, DLBCL, and MZL was evaluated in relation to the presence of any anti-ENA or anti-dsDNA, as well as anti-SSA and anti-dsDNA individually, using logistic regression, adjusting for age, race, and gender. For cells with small sample sizes (*n* < 5) amongst cases or controls, we utilized Fisher’s exact tests to determine ORs and 95% CI for the relationship with NHL subtypes. The prevalence of other anti-ENAs (e.g., anti-SSB, anti-Sm, anti-nRNP) was too small for meaningful analysis by themselves.

## 3. Results

The study participants were predominantly white, non-Hispanic (93.5%), with a median age of 71 (IQR: 66–76) in cases and 71 (IQR: 66–76) in controls (Table 1). Comparing baseline characteristics between common NHL subtypes and controls, MZL and CLL cases differed from controls with regards to race (*p* = 0.04 and *p* = 0.03 respectively), and FL cases were less likely to smoke compared to controls, whereas MZL cases were more likely to smoke (*p* = 0.009 and *p* = 0.02 respectively). However, after utilizing a Bonferroni corrected alpha level (α = 0.0012) to compensate for the number of tests conducted, none of these differences in baseline characteristics between NHL subtypes and the controls remained statistically significant, and overall, controls were similar to cases with regard to age and gender. The median time from blood draw to NHL diagnosis for cases was 6.95 years (interquartile range (IQR): 4.04–9.71 years). Approximately, 13% of our study population tested positive for ANA, including 12.2% of controls and 14.1% of cases.

Overall, there was no association between ANA positivity and the risk of B-cell NHL (OR: 1.21, 95% CI: 0.91–1.63), T-cell NHL (OR: 0.71, 95% CI: 0.21–2.40), or NHL generally (OR: 1.18, 95% CI: 0.88–1.58) (Table 2, Supplementary Table S1); however, differences were observed by subtype (*p* = 0.04, Table 2, Supplementary Table S2). A positive ANA test was associated with an increased risk of DLBCL (OR: 1.83, 95% CI: 1.15–2.91, *p* = 0.01). The exclusion of participants who self-reported an auto-immune disease (*n* = 98) of ulcerative colitis, Crohn’s disease, or rheumatoid arthritis did not substantially alter the risk of DLBCL (OR: 1.91, 95% CI: 1.18–3.08, *p* = 0.009) or NHL overall (OR: 1.20, 95% CI: 0.89–1.62). Similarly, the exclusion of those testing positive for biomarkers of rheumatoid arthritis (RF-IgA, RF-IgM, or anti-CCP3) did not significantly alter the association between ANA and DLBCL (OR: 1.93, 95% CI: 1.11–3.36) or NHL (OR: 1.13, 95% CI: 0.80–1.61) risk. When the analysis was restricted to only matched cases and controls and analyzed with unconditional or conditional logistic regression, the OR for DLBCL was slightly smaller in magnitude and no longer statistically significant with the smaller sample size (OR: 1.49, 95% CI: 0.80–2.76 and OR: 1.53, 95% CI: 0.80–2.94, respectively, Supplementary Table S2).

**Figure 1:**
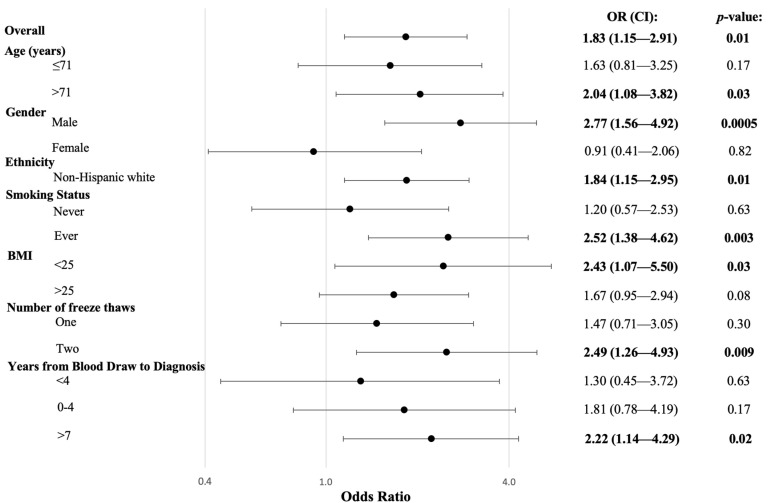
Risk of DLBCL Associated with the Presence of ANA Stratified by Demographic and Risk Factors. Logistic regression was utilized to produce OR, 95% CIs, and p-values, adjusted for age, gender, and race. Interactions were not significant, except for gender (*p* = 0.03).

The relationship between ANA positivity and DLBCL risk was further investigated in stratified analyses with potential effect modifiers (Figure 1). There was a strong positive association between ANA and DLBCL risk among males (OR: 2.77, 95% CI: 1.56–4.92, *p* = 0.0005) but no association among females (OR: 0.91, 95% CI: 0.41–2.06), and the interaction between gender and ANA was significant (*p*_interaction_ = 0.03). ANA positivity was associated with a higher risk of DLBCL among ever-smokers (OR: 2.52, 95% CI: 1.38–4.62, *p* = 0.003) compared to never-smokers (OR: 1.20, 95% CI: 0.57–2.53), but the interaction was not significant (*p*_interaction_ = 0.12). Similarly, a positive ANA test was associated with a slightly greater risk of DLBCL among those with blood draws four or more years prior to diagnosis (OR: 1.99, 95% CI: 1.19–3.34, *p* = 0.009), compared to those with blood draws closer to diagnosis (OR: 1.30, 95% CI: 0.45–3.72), but the interaction was not significant (*p*_interaction_ = 0.54).

Among participants who tested positive for ANA, 13.0% also tested positive for at least one extractable nuclear antigen antibody (anti-ENA) or anti-dsDNA, including 18.0% of cases and 7.1% of controls. Those who were positive for at least one other autoantibody (anti-ENA or anti-dsDNA) were discovered to have a roughly three-fold increase in the risk of developing NHL overall compared to those who tested negative (OR: 2.93, 95% CI: 1.18–7.28, *p* = 0.02, Table 3) or those who were untested (OR: 3.01, 95% CI: 1.27–7.15, *p* = 0.01, Supplementary Table S3). The results were similar for B-cell NHL (OR: 2.57, 95% CI: 1.02–6.47, *p* = 0.045). Although the number of cases for specific subtypes was small, a positive test for at least one other autoantibody (anti-ENA or anti-dsDNA) was associated with a significantly increased risk of developing DLBCL or MZL compared to those who tested negative (OR: 3.51, 95% CI: 1.02–12.0, *p* = 0.046 and OR: 8.86, 95% CI: 1.26–62.0, *p* = 0.015, respectively). The increased risk of DLBCL and MZL observed in those with at least one positive test appeared to be driven by a positive anti-SSA test (Table 3).

## 4. Discussion

In this nested case-control study of autoantibodies, we discovered evidence supporting the hypothesis that immune dysregulation and autoimmunity years prior to diagnosis are associated with an increased risk of NHL with heterogeneity among subtypes. We observed that a positive ANA test was associated with an increased risk of developing DLBCL. Stratified analyses suggested that the increased risk may be stronger for males, and the interaction was statistically significant (*p* = 0.03). Although we did not observe evidence of an association between ANA positivity and other NHL subtypes or NHL as a whole, we discovered that testing positive for at least one other autoimmune antibody (e.g., anti-ENA or anti-dsDNA) was associated with an increased risk of NHL, particularly DLBCL and MZL.

Our study is consistent with epidemiologic studies suggesting that autoimmune disease is associated with some, but not all, NHL subtypes. Previous research has suggested that there is a positive but heterogeneous relationship between autoimmune diseases and the risk of NHL, with some NHL subtypes showing a strong relationship and others a weak or null association [8]. Although some epidemiological studies have shown a positive association between one or more autoimmune diseases and the risk of developing CLL [4,23,24], other large-scale studies have found no such relationship [25,26]. Autoimmune diseases have been consistently associated with an increased risk of DLBCL and MZL in epidemiologic studies [27,28]. An early pooled analysis from the InterLymph Consortium reported that autoimmune diseases, such as lupus and hemolytic anemia, were associated with an increased risk of developing DLBCL [29]. Similarly, a small case-control study found those with systemic lupus erythematosus had a higher risk of developing hematological malignancies, with almost half consisting of NHL patients, the majority of whom were diagnosed with DLBCL [30]. A follow-up pooled analysis of 19 case-control studies from the InterLymph Consortium found that B-cell activating autoimmune disorders were associated with an increased risk of developing DLBCL [27] and MZL [28]. A large population-based study using the SEER-Medicare database found that DLBCL was significantly associated with nine autoimmune conditions, including rheumatoid arthritis, Sjögren’s syndrome, sarcoidosis, and aplastic anemia, and MZL was associated with three autoimmune conditions, namely Sjögren’s syndrome, systemic lupus erythematosus, and hemolytic anemia [4].

Our study is largely consistent with smaller retrospective studies of serum autoimmune antibodies and NHL risk. One hospital-based case-control study reported a higher prevalence of ANA as well as other autoimmune antibodies among lymphoma patients compared to healthy controls [29]. A retrospective cohort study found that 77% of non-Hodgkin lymphoma patients had autoantibodies at the time of diagnosis, with 44% of those testing positive for ANA [31]. Although we did not observe an increased risk of NHL overall with ANA in our study, we did observe a higher risk of NHL with the presence of at least one other autoimmune antibody. Similar to the results of our study, a small hospital-based study reported that antinuclear autoantibodies were more prevalent in DLBCL cases than in healthy controls [32], and a hospital-based study of antinuclear autoantibodies found that DLBCL patients were more likely to be positive for ANA, anti-Jo-1, anti-ssDNA, and perinuclear anti-neutrophil cytoplasmic antibody compared to NHL patients with other subtypes [33]. In hospital-based studies, the majority of lymphoma patients with antinuclear autoantibodies did not have a diagnosis or symptoms of autoimmune disease, suggesting the presence of ANA may be reflective of other immune dysregulation as opposed to an existing autoimmune disease [20,32]. Other studies have reported that ANA positivity is not necessarily predictive of autoimmune disease with low positive predictive value in clinical settings [13,14].

In our study, although the numbers were small, we found that the presence of anti-SSA antibodies was associated with an increased risk of DLBCL and MZL. Anti-SSA/SSB antibodies are often found in patients with Sjögren’s syndrome, as well as other autoimmune diseases [34]. Patients with Sjögren’s syndrome are at an increased risk of developing lymphoma. Two studies of Sjögren’s syndrome patients have shown that patients with anti-SSA/SSB antibodies were significantly more likely to develop lymphoma than those without anti-SSA/SSB antibodies [35,36]. These studies, in conjunction with our study, suggest that the presence of anti-SSA/SSB antibodies may be an independent risk factor for NHL, particularly DLBCL and MZL.

Our study includes several limitations. The participants in our study were predominantly white, non-Hispanic individuals, limiting the generalizability of our results to other racial/ethnic populations. Additional studies are needed in non-white populations. Although our results included different subtypes, we had limited power to evaluate the less common subtypes, such as MZL, and were unable to evaluate rare subtypes, such as Waldenström’s macroglobulinemia. Pooled analyses across cohort studies would provide greater power to investigate the association with rare NHL subtypes. Although excluding individuals with reported diagnoses of autoimmune diseases of ulcerative colitis, Crohn’s disease, or rheumatoid arthritis or removal of those with positive biomarkers for rheumatoid arthritis (anti-CCP3, RF-IgA, and RF-IgM) did not significantly change the risk of NHL or DLBCL, we did not have data for all autoimmune diseases, medications for autoimmune diseases, or immunosuppressive treatment. It is possible that some participants were on immunosuppressive treatment for other conditions at the time of blood draw; however, individuals undergoing treatment for cancer were excluded from the trial, and PLCO participants have been reported to be healthier than the general population [37]. In addition, individuals could have had other autoimmune diseases that were not captured by our baseline questionnaire, and we cannot rule out the possibility that the results observed were driven by the presence of other autoimmune diseases. However, the exclusion of common autoimmune diseases in our analyses likely captured the majority of individuals with autoimmune conditions. Finally, the sample size was a limiting factor in our analyses of anti-SSA. Additional nested case-control studies are needed to confirm the association between anti-SSA and DLBCL and MZL.

The strengths of our study include the large sample of blood specimens from NHL cases collected years prior to diagnosis and the ability to evaluate common NHL subtypes. DLBCL represents one of the largest subtypes of incident NHL [38]. Our results help clarify the etiologic role of autoantibodies in DLBCL risk, showing that autoimmunity years prior to diagnosis is a risk factor for DLBCL. In addition, specific autoimmune antibodies, such as anti-SSA, may be important risk factors for disease. Our large, nested case-control study elucidates the relationship between autoantibody levels and the risk of developing NHL, showing the heterogeneity in risk by subtype. With blood specimens collected as part of the PLCO Cancer Screening Trial, our nested case-control study allows us to establish the presence of ANA and anti-SSA years prior to diagnosis in cases and suggests that the associations are unlikely to be attributable to reverse causation. Although early disease may have been present in some subjects, the fact that the association between ANA and DLBCL was stronger for cases with blood draw > 4 years suggests that early disease is unlikely to explain the association between ANA and DLBCL risk. As our study used collected specimens from years prior to diagnosis, our results are also not subject to the reporting and selection biases that may occur in retrospective studies.

## 5. Conclusions

Given the rise in autoimmunity in the U.S. [11], health risks associated with autoimmunity are an important public health concern. Our study provides evidence that autoimmunity years prior to diagnosis is associated with an increased risk of DLBCL and that the presence of other specific autoimmune antibodies, such as anti-SSA, may be a risk factor for developing DLBCL and MZL. Additional large, nested case-control studies are needed to confirm our results. Further exploration of autoimmune antibodies with specific DLBCL subtypes and prognosis is warranted.

## Figures and Tables

**Table 1:** Baseline characteristics of the NHL cases and controls in PLCO Cancer Screening Trial.

	DLBCL	FL	MZL	CLL	MM	Other B-Cell	All B-Cell Lymphoma	All T-Cell Lymphoma	Controls
Characteristic	*n* = 147	*n* = 92	*n* = 28	*n* = 223	*n* = 176	*n* = 111	*n* = 777	*n* = 34	*n* = 809
Age at dx/selection, *median (IQR)*	72 (66–77)	69 (66–74)	70 (66–76)	70 (65–75)	71 (67–76)	72 (67–76)	71 (66–76)	71 (66–74)	71 (66–76)
Gender, *n (%)*									
Male	93 (63.3%)	48 (52.2%)	15 (53.6%)	131 (58.7%)	109 (61.9%)	71 (64.0%)	467 (60.1%)	22 (64.7%)	487 (60.2%)
Female	54 (36.7%)	44 (47.8%)	13 (46.4%)	92 (41.3%)	67 (38.1%)	40 (36.0%)	310 (39.9%)	12 (35.3%)	322 (39.8%)
Race, *n (%)*									
White, non-Hispanic	141 (95.9%)	88 (95.7%)	24 (85.7%)	217 (97.3%)	161 (91.5%)	99 (89.2%)	730 (94.0%)	30 (88.2%)	756 (93.5%)
Black, non-Hispanic	2 (1.4%)	0 (0%)	3 (10.7%)	5 (2.2%)	7 (4.0%)	3 (2.7%)	20 (2.6%)	1 (2.9%)	21 (2.6%)
Other	4 (2.7%)	4 (2.7%)	1 (3.6%)	1 (0.5%)	8 (4.5%)	9 (8.1%)	27 (3.5%)	3 (8.8%)	32 (4.0%)
Family hx of hem cancer, *n (%)*									
Yes	19 (13.0%)	12 (13.5%)	3 (10.7%)	25 (11.5%)	18 (10.2%)	13 (11.7%)	90 (11.8%)	2 (5.9%)	72 (9.0%)
No	127 (87.0%)	77 (86.5%)	25 (89.3%)	193 (88.5%)	158 (89.8%)	96 (86.5%)	676 (88.3%)	32 (94.1%)	729 (91.0%)
Smoking status, *n (%)*									
Never	71 (48.3%)	58 (63.7%)	10 (35.7%)	108 (48.4%)	90 (51.1%)	49 (44.1%)	386 (49.7%)	14 (41.2%)	398 (49.2%)
Former	61 (41.5%)	24 (26.4%)	12 (42.9%)	94 (42.2%)	79 (44.9%)	48 (43.2%)	318 (41.0%)	17 (50.0%)	348 (43.0%)
Current	15 (10.2%)	9 (9.9%)	6 (21.4%)	21 (9.4%)	7 (4.0%)	14 (12.6%)	72 (9.3%)	3 (8.8%)	63 (7.8%)
BMI, *mean ± SD*	27.7 ± 4.3	27.3 ± 5.4	28.5 ± 4.5	27.4 ± 4.4	26.8 ± 5.3	27.3 ± 4.6	26.7 ± 4.7	27.0 ± 3.2	27.3 ± 4.5

Abbreviations: DLBCL = Diffuse Large B-Cell Lymphoma; FL = Follicular Lymphoma; MZL = Marginal Zone Lymphoma; CLL = Chronic Lymphocytic Leukemia; MM = Multiple Myeloma; Other B-cell includes other less common B-cell subtypes; All B-Cell Lymphomas includes all B-cell subtypes as well as B-cell, NOS; All T-Cell Lymphomas includes all T-cell subtypes as well as T-cell, NOS; Hx = history; Hem = hematopoietic; BMI = body mass index.

**Table 2:** Risk of NHL by the presence of serum antinuclear antibodies (ANA).

		NHL Overall		B-Cell NHL		DLBCL		MZL		CLL		FL		MM	
	No. Cntrl	No. Cases	OR (95% CI)	No. Cases	OR (95% CI)	No. Cases	OR (95% CI)	No. Cases	OR (95% CI)	No. Cases	OR (95% CI)	No. Cases	OR (95% CI)	No. Cases	OR (95% CI)
ANA															
negative	710	715	*ref*	665	*ref*	118	*ref*	23	*ref*	193	*ref*	79	*ref*	154	*ref*
positive	99	117	1.18 (0.88–1.58)	112	1.21 (0.91–1.63)	29	1.83 (1.15–2.91)	5	1.49 (0.55–4.05)	30	1.07 (0.69–1.67)	13	1.10 (0.58–2.06)	22	1.05 (0.64–1.72)

Abbreviations: NHL = Non-Hodgkin lymphoma; B-cell NHL includes all B-cell NHL subtypes; DLBCL = Diffuse Large B-Cell Lymphoma; FL = Follicular Lymphoma; MZL = Marginal Zone Lymphoma; CLL = Chronic Lymphocytic Leukemia; MM = Multiple Myeloma; cntrl = controls.

**Table 3:** Risk of NHL by the presence of serum anti-ENAs and anti-dsDNA.

		NHL Overall		B-Cell NHL		DLBCL		MZL		CLL		FL		MM	
	No. Cntrl	No. Cases	OR (95% CI)	No. Cases	OR (95% CI)	No. Cases	OR (95% CI)	No. Cases	OR (95% CI)	No. Cases	OR (95% CI)	No. Cases	OR (95% CI)	No. Cases	OR (95% CI)
Anti-ENA or Anti-dsDNA															
Negative	93	95	*reference*	93	*reference*	22	*reference*	3	*reference*	26	*reference*	13	*reference*	20	*reference*
Positive	7	21	2.93 (1.18–7.28)	18	2.57 (1.02–6.47)	6	3.51 (1.02–12.0)	2	8.86 (1.26–62.0)	4	2.04 (0.56–7.52)	0	x	2	1.33 (0.26–6.88)
Anti-dsDNA															
negative	96	104	*reference*	101	*reference*	26	*reference*	5	*reference*	26	*reference*	13	*reference*	21	*reference*
Positive	4	12	2.70 (0.83–8.79)	10	2.34 (0.70–7.78)	2	1.85 (0.32–10.6)	0	x	4	3.69 (0.86–15.8)	0	x	1	1.14 (0.12–10.8)
Anti-SSA															
Negative	97	106	*reference*	103	*reference*	24	*reference*	3	*reference*	30	*reference*	13	*reference*	21	*reference*
Positive	3	10	3.12 (0.83–11.7)	8	2.59 (0.67–10.1)	4	5.39 (1.13–25.7)	2	21.6 (2.57–181)	0	x	0	x	1	1.54 (0.15–15.5)

Abbreviations: NHL = Non-Hogkin Lymphoma; DLBCL = Diffuse Large B-Cell Lymphoma; MZL = Marginal Zone Lymphoma; CLL = Chronic Lymphocytic Leukemia; FL = Follicular Lymphoma; MM = Multiple Myeloma; cntrl = controls; Anti-ENA = extractable nuclear antigen antibodies; Anti-dsDNA = anti-double stranded deoxyribonucleic acid antibodies; Anti-SSA = anti-Sjögren’s-syndrome type A antibodies. **Note:** Participants testing positive for ANA were tested for extractable nuclear antigen antibodies (anti-ENAs), including anti-Sjogren’s-syndrome type A (anti-SSA), and anti-double stranded deoxyribonucleic acid (anti-dsDNA) antibodies. Anti-ENA or anti-dsDNA positive includes those who were positive for anti-dsDNA (*n* = 15), positive for anti-dsDNA and anti-SSA (*n* = 1), positive for anti-SSA (*n* = 9), and positive for anti-SSA and anti-SSB (*n* = 3). Fisher’s exact test was utilized for cells with *n* < 5.

## Data Availability

Data used in this study are available for research purposes upon request and approval from the PLCO Cancer Data Access System.

## Supplementary Materials

The following supporting information can be downloaded at: https://www.mdpi.com/article/10.3390/cancers15215231/s1, Table S1. Risk of B-cell, T-cell, and overall NHL associated with ANA status using unconditional logistic regression and all controls, unconditional logistic regression and matched controls, and conditional logistic regression and matched controls. Table S2. Risk of NHL subtypes associated with ANA status using unconditional logistic regression and all controls, unconditioned logistic regression and matched controls, and conditional logistic regression and matched controls. Table S3. Risk of NHL by the presence of serum anti-ENAs and anti-dsDNA.
