# Supplementary material for: Antinuclear Antibodies Are Associated with an Increased Risk of Diffuse Large B-Cell Lymphoma
Source: Cancers (Basel). 2023 Oct 31;15(21):5231. doi: 10.3390/cancers15215231 (PMC10647241; doi:10.3390/cancers15215231)
Supplement: Supplementary file 1 [file cancers-15-05231-s001.zip › cancers-2628886-supplementary.pdf]

Supplementary Table 1: Risk of B-cell, T-cell, and NHL associated with ANA status using unconditional logistic regression and all controls, unconditional logistic regression and matched controls, and conditional logistic regression and matched controls

	B-Cell			T-Cell			ALL NHL		
**Unconditional (All subjects)**	**cases**	**ctrls**	**OR (CI)**	**cases**	**ctrls**	**OR (CI)**	**cases**	**ctrls**	**OR (CI)**
ANA negative	665	710	*reference*	31	710	*reference*	715	710	*reference*
ANA positive	112	99	1.21 (0.91-1.63)	3	99	0.71 (0.21-2.40)	117	99	1.18 (0.88-1.58)
**Unconditional (Matched cases and controls only)**									
ANA negative	646	664	*reference*	30	28	*reference*	694	710	*reference*
ANA positive	110	92	1.23 (0.91-1.66)	3	5	0.54 (0.12-2.55)	115	99	1.19 (0.89-1.59)
**Conditional (Matched cases and controls only)**									
ANA negative	646	664	*reference*	30	28	*reference*	694	710	*reference*
ANA positive	110	92	1.25 (0.92-1.70)	3	5	0.60 (0.14-2.51)	115	99	1.20 (0.89-1.62)

Abbreviations: NHL = Non-Hodgkin lymphoma; B-Cell NHL includes all B-cell NHL subtypes as well as B-cell, NOS; T-Cell NHL includes all T-cell NHL subtypes as well as T-cell, NOS; ANA = Antinuclear antibodies, OR = Odds ratio, CI = Confidence interval.

Supplementary Table 2: Risk of specific NHL subtypes associated with ANA using unconditional logistic regression and all controls, unconditional logistic regression and matched controls, and conditional logistic regression and matched controls

	DLBCL			FL			MZL			CLL			MM			Other B-Cell		
**Unconditional (All subjects)**	**cases**	**ctrls**	**OR (CI)**	**cases**	**ctrls**	**OR (CI)**	**cases**	**ctrls**	**OR (CI)**	**cases**	**ctrls**	**OR (CI)**	**cases**	**ctrls**	**OR (CI)**	**cases**	**ctrls**	**OR (CI)**
ANA negative	118	710	*reference*	79	710	*reference*	23	710	*reference*	193	710	*reference*	154	710	*reference*	98	710	*reference*
ANA positive	29	99	1.83 (1.15-2.91)	13	99	1.10 (0.58-2.06)	5	99	1.49 (0.55-4.05)	30	99	1.07 (0.69-1.67)	22	99	1.05 (0.64-1.72)	13	99	0.99 (0.53-1.84)
**Unconditional (Matched cases and controls only)**																		
ANA negative	115	123	*reference*	74	74	*reference*	22	25	*reference*	188	195	*reference*	152	150	*reference*	95	97	*reference*
ANA positive	29	21	1.49 (0.80-2.76)	13	13	1.00 (0.43-2.34)	5	2	3.00 (0.50-18.0)	30	23	1.36 (0.76-2.44)	21	23	0.90 (0.47-1.71)	12	10	1.23 (0.50-2.99)
**Conditional (Matched cases and controls only)**																		
ANA negative	115	123	*reference*	74	74	*reference*	22	25	*reference*	188	195	*reference*	152	150	*reference*	95	97	*reference*
ANA positive	29	21	1.53 (0.80-2.94)	13	13	1.00 (0.43-2.31)	5	2	2.50 (0.49-12.9)	30	23	1.44 (0.76-2.72)	21	23	0.90 (0.48-1.70)	12	10	1.25 (0.49-3.17)

Abbreviations: NHL = Non-Hodgkin Lymphoma; DLBCL = Diffuse Large B-Cell Lymphoma; FL = Follicular Lymphoma; MZL = Marginal Zone Lymphoma; CLL = Chronic Lymphocytic Leukemia; MM = Multiple Myeloma; ANA = Antinuclear antibodies.

Supplementary Table 3: Risk of NHL by the Presence of Serum Anti-ENAs and Anti-dsDNA

			NHL Overall		B-Cell NHL			DLBCL			MZL			CLL			FL			MM		
	control	case	OR (95% CI)*	OR (95% CI)*	case	OR (95% CI)*	OR (95% CI)*	case	OR (95% CI)*	OR (95% CI)*	case	OR (95% CI)*	OR (95% CI)*	case	OR (95% CI)*	OR (95% CI)*	case	OR (95% CI)*	OR (95%)	case	OR (95% CI)*	OR (95% CI)*
**Anti-ENA or Anti-dsDNA****																						
untested	709	716	*ref*	.	666	*ref*	.	119	*ref*	.	23	*ref*	.	193	*ref*	.	79	*ref*	.	154	*ref*	.
negative	93	95	1.02 (0.75-1.38)	*ref*	93	1.07 (0.79-1.45)	*ref*	22	1.46 (0.88-2.42)	*ref*	3	0.96 (0.28-3.28)	*ref*	26	0.99 (0.62-1.59)	*ref*	13	1.17 (0.62, 2.21)	*ref*	20	1.01 (0.60-1.69)	*ref*
positive	7	21	**3.01 (1.27-7.15)**	**2.93 (1.18-7.28)**	18	**2.76 (1.15-6.66)**	**2.57 (1.02-6.47)**	6	**5.15 (1.69-15.7)**	**3.51 (1.02-12.0)**	2	**8.81 (1.73-44.8)**	**8.86 (1.26-62.0)**	4	2.10 (0.61-7.25)	2.04 (0.56-7.52)	0	x	x	2	1.32 (0.27-6.39)	1.33 (0.26-6.88)
**Anti-dsDNA**																						
untested	709	716	*ref*	.	666	*ref*	.	119	*ref*	.	23	*ref*	.	193	*ref*	.	79	*ref*	.	154	*ref*	.
negative	96	104	1.08 (0.80-1.45)	*ref*	101	0.91 (0.71-1.16)	*ref*	26	**1.67 (1.03-2.70)**	*ref*	5	1.53 (0.56-4.17)	*ref*	26	0.96 (0.61-1.54)	*ref*	13	1.13 (0.60, 2.13)	*ref*	21	1.03 (0.62-1.70)	*ref*
positive	4	12	3.03 (0.97-9.47)	3.00 (0.96-9.36)	10	0.85 (0.64-1.12)	2.34 (0.70-7.78)	2	2.98 (0.54-16.5)	1.85 (0.32-10.6)	0	x	x	4	3.67 (0.91-14.8)	3.69 (0.86-15.8)	0	x	x	1	1.15 (0.13-10.4)	1.14 (0.12-10.8)
**Anti-SSA**																						
untested	709	716	*ref*	.	666	*ref*	.	119	*ref*	.	23	*ref*	.	193	*ref*	.	79	*ref*	.	154	*ref*	.
negative	97	106	1.09 (0.81-1.46)	*ref*	103	1.14 (0.84-1.53)	*ref*	24	1.52 (0.93-2.50)	*ref*	3	0.92 (0.27-3.16)	*ref*	30	1.09 (0.70-1.70)	*ref*	13	1.12 (0.60, 2.11)	*ref*	21	1.02 (0.61-1.69)	*ref*
positive	3	10	3.33 (0.91-12.2)	3.12 (0.83-11.7)	8	2.83 (0.75-10.7)	2.59 (0.67-10.1)	4	**7.94 (1.76-35.9)**	**5.39 (1.13-25.7)**	2	**20.6 (3.27-129)**	**21.6 (2.57-181)**	0	x	x	0	x	x	1	1.54 (0.16-14.9)	1.54 (0.15-15.5)

Abbreviations: NHL = Non-Hodgkin Lymphoma; DLBCL = Diffuse Large B-Cell Lymphoma; MZL = Marginal Zone Lymphoma; CLL = Chronic Lymphocytic Leukemia; FL = Follicular Lymphoma; MM = Multiple Myeloma; cntrl = controls; Anti-ENA = extractable nuclear antigen antibodies; Anti-dsDNA = anti-double stranded deoxyribonucleic acid antibodies; Anti-SSA = anti- Sjögren's -syndrome type A antibodies. **Note:** Participants testing positive for ANA were tested for extractable nuclear antigen antibodies (anti-ENAs), including anti-Sjogren's-syndrome type A (anti-SSA), and anti-double stranded deoxyribonucleic acid (anti-dsDNA) antibodies. Anti-ENA or anti-dsDNA positive includes those who were positive for anti-dsDNA (n=15), positive for anti-dsDNA and anti-SSA (n=1), positive for anti-SSA (n=9), and positive for anti-SSA and anti-SSB (n=3). Fisher's exact test was utilized for cells with n<5.
